# Guidance for Systematic Integration of Undernutrition in Attributing Cause of Death in Children

**DOI:** 10.1093/cid/ciab851

**Published:** 2021-12-15

**Authors:** Christina R Paganelli, Nicholas Kassebaum, Kathleen Strong, Parminder S Suchdev, Wieger Voskuijl, Quique Bassat, Dianna M Blau, Donna M Denno

**Affiliations:** RTI International, Seattle, Washington, USA; Department of Health Metrics Sciences, University of Washington, Seattle, Washington, USA; Department of Global Health, University of Washington, Seattle, Washington, USA; Department of Anesthesiology and Pain Medicine, University of Washington, Seattle, Washington, USA; Department of Maternal, Newborn, Child and Adolescent Health and Aging, World Health Organization, Geneva, Switzerland; Department of Pediatrics and Emory Global Health Institute, Emory University, Atlanta, Georgia, USA; University of Amsterdam, Amsterdam, the Netherlands; Amsterdam Centre for Global Health, Emma Children’s Hospital, Amsterdam University Medical Centres, Amsterdam, the Netherlands; Amsterdam Institute for Global Health and Development, Amsterdam University Medical Centres, Amsterdam, the Netherlands; The Childhood Acute Illness & Nutrition Network, Nairobi, Kenya; ISGlobal, Hospital Clínic–Universitat de Barcelona, Barcelona, Spain; Centro de Investigação em Saúde de Manhiça (CISM), Maputo, Mozambique; ICREA, Pg. Lluís Companys 23, 08010 Barcelona, Spain; Pediatrics Department, Hospital Sant Joan de Déu, Universitat de Barcelona, Esplugues, Barcelona, Spain; Consorcio de Investigación Biomédica en Red de Epidemiología y Salud Pública (CIBERESP), Madrid, Spain; Centers for Disease Control and Prevention, Atlanta, Georgia, USAand; Department of Global Health, University of Washington, Seattle, Washington, USA; The Childhood Acute Illness & Nutrition Network, Nairobi, Kenya; Department of Pediatrics, University of Washington, Seattle, Washington, USA

**Keywords:** Undernutrition, child mortality surveillance, severe wasting, cause of death, minimally invasive tissue sampling

## Abstract

Minimally invasive tissue sampling (MITS) is increasingly being used to better understand causes of death in low-resource settings. Undernutrition (eg, wasting, stunting) is prevalent among children globally and yet not consistently coded or uniformly included on death certificates in MITS studies when present. Consistent and accurate attribution of undernutrition is fundamental to understanding its contribution to child deaths. In May 2020, members of the MITS Alliance Cause of Death Technical Working Group convened a panel of experts in public health, child health, nutrition, infectious diseases, and MITS to develop guidance for systematic integration of undernutrition, as assessed by anthropometry, in cause of death coding, including as part of the causal chain or as a contributing condition, in children <5 years of age. The guidance presented here will support MITS and other researchers, public health practitioners, and clinicians with a systematic approach to assigning and interpreting undernutrition in death certification.

Undernutrition has a profound impact on child morbidity and mortality and remains prevalent in low- and middle-income countries (LMICs) [1]. Globally, 6.7% of children in 2020 had a weight-for-height/length *z* score below −2, only modestly declined from 10% in 2005 [2, 3]. Children with a weight-for-height/length *z* score below −3 or between − 3 and less than −2 have an 11.6- or 3.4-fold increased mortality risk, respectively [1, 4].

Postmortem minimally invasive tissue sampling (MITS) consists of gross visualization, anthropometric measurements, and transcutaneous needle sampling of blood, cerebrospinal fluid, and organs, such as brain, lungs, liver, and heart. Samples undergo histopathological and microbiological assessments to support cause of death determination. Compared with standard autopsy, MITS is less invasive, requires fewer resources, can be performed more quickly, and may be more acceptable to families [5, 6]. MITS has been validated compared with standard autopsy, and MITS studies are increasingly becoming important sources of data on direct and underlying causes of child death, especially in LMICs [7–9].

Established in 2017, the MITS Surveillance Alliance (https://mitsalliance.org/) is a global multidisciplinary consortium of researchers convened to support knowledge sharing and expanded use of MITS, including through technical working groups. The Cause of Death technical working group works to standardize approaches for assigning cause of death as part of MITS studies. Members of this group observed that although anthropometry is a standard component of MITS procedures, undernutrition across MITS studies was not consistently coded or even uniformly included in death certification or cause of death reports. Inaccurate attribution of undernutrition as either an underlying or a contributing cause of death can result in both underestimation and overestimation of the role of undernutrition in child deaths. Improved precision in classification and attribution of undernutrition as a cause of death will provide a more comprehensive understanding of its role, direct or indirect, in all-cause child mortality, which can lead to improved and novel interventions and, ultimately, lives saved. A need for guidance on the systematic integration of undernutrition as contributing conditions or the in the causal chain of events leading to death in children <5 years of age was identified.

## MITS ANTHROPOMETRY

Anthropometric measurements typically assessed during MITS procedures include weight, mid-upper arm circumference (MUAC), and recumbent length (in life, standing height is recommended for those ≥2 years of age who are ambulatory) [10]. Weight-for-length *z* (WLZ), length-for-age *z* (LAZ), and weight-for-age *z* (WAZ) scores are calculated based on World Health Organization (WHO) growth standards for children <5 years of age [1, 2, 11–13] (Table 1). Wasting, a form of undernutrition characterized by thinness, and often referred to as “acute malnutrition,” is defined by severely low MUAC (among children ≥6 months of age) or WLZ scores or the presence of bipedal nutritional edema. Underweight, defined as WAZ score <–2, is often used as a measure of poor anthropometric status among infants <6 months old as it is considered a better predictor of mortality risk among this age group, compared with WLZ scores [14]. Stunting, defined by LAZ score below −2, is caused by multiple factors (eg, recurrent infections, poor feeding practices, food insecurity, recurrent infections, and other causes of systemic inflammation) and is often used as a population-level indicator of chronic deprivation [15].

**Table 1.** Undernutrition Terms and Definitions

Term	Definition
Malnutrition	Includes undernutrition and overnutrition
Undernutrition	Wasting, stunting, underweight, or micronutrient deficiencies^a^
Severe wasting	MUAC <11.5cm (among children ≥6 mo old^b^), WLZ score <−3, or presence of bipedal nutritional edema
Marasmus	Form of severe wasting characterized by low MUAC or low WLZ score
Kwashiorkor	Form of severe wasting characterized by bipedal nutritional edema
Marasmic kwashiorkor	Form of severe wasting characterized by bipedal nutritional edema and low MUAC or WLZ score
Moderate wasting	MUAC < 12.5cm but ≥11.5cm (among children ≥6 mo old^b^) or WLZ score below −2 but above or equal to −3
Underweight	WAZ score <−2
Stunting	LAZ score < −2
LBW	BW <2500 g
SGA	BW-for-GA <10th centile
Prematurity	Live birth before 37 weeks gestation

Abbreviations: BW, birth weight; GA, gestational age; LAZ, length-for-age *z*; LBW, low BW; MUAC, mid-upper arm circumference; SGA, small for GA; WAZ, weight-for-age *z*; WLZ, weight-for-length *z*.

a Micronutrient deficiencies were outside the scope of this guidance.

b MUAC reference standards have yet to be established for children <6 months of age.

Antemortem anthropometric data are often limited, absent, or not measured or recorded with sufficient precision. When available, antemortem anthropometric data should be evaluated along with postmortem data, which should be measured per protocol to optimize accuracy (see objective 3). Antemortem assessments can contextualize postmortem data and are the only option to inform growth trajectories, including changes in LAZ, WAZ, and WLZ scores [16].

## OBJECTIVES AND APPROACH

Three members of the MITS Alliance Cause of Death technical working group convened a multidisciplinary panel of experts in public health, child health, child nutrition, infectious disease, and MITS. The group met via videoconference 10 times from May 2020 to May 2021. The following objectives were delineated and provided structure for guidance development:

- Objective 1: Establish parameters for when severe and moderate wasting and stunting in children 1–59 months old should be included in death certification and under what circumstances the condition should be included in part 1 (causal chain) or in part 2 (contributing condition).
- Objective 2: Generate a framework for how to account for low birth weight (LBW), prematurity, and small for gestational age (SGA) as contributing or causal conditions of wasting in deaths among 1–3-month-old infants.
- Objective 3: Standardize methods for using postmortem anthropometric assessments, especially in the context of limited antemortem data, to identify wasting and stunting.

We strove to align guidance development with the *International Statistical Classification of Diseases and Related Health Problems Tenth Revision* (*ICD-10*). Although the 11^th^ revision, *ICD-11,* has been released, widespread implementation is not anticipated until 2022. The panel therefore chose to use *ICD-10* to support immediate application in MITS studies. However, we anticipate full applicability of the guidance with *ICD-11*; sample code mapping is provided in Table 2.

**Table 2.** Mapping Conditions to *ICD-10* and *ICD-11* Codes

Undernutrition Conditions	*ICD-10* Code	*ICD-11* Code
Severe wasting	E40–E43 E40 Kwashiorkor E41 Nutritional marasmus E42 Marasmic kwashiorkor E43 Severe wasting, not otherwise categorized	5B51^a^ Wasting in infants, children, or adolescents XS25 (severe) or 5B52^a^ Acute malnutrition in infants, children, or adolescents XS25 (severe)
	B22.2 Wasting syndrome in a child living with HIV infection^b^	5B51^a^ Wasting in infants, children, or adolescents XS25 (severe) or 5B52^a^ Acute malnutrition in infants, children, or adolescents XS25 (severe)
Moderate wasting	E44.0	5B51^a^ Wasting in infants, children, or adolescents XS0T (moderate) or 5B52^a^ Acute malnutrition in infants, children, or adolescents XS0T (moderate)
	B22.2 HIV disease resulting in wasting syndrome^b^	
Stunting	E45	5B53^a^ Stunting in infants, children, or adolescents
SGA	P05 Slow fetal growth and fetal malnutrition	KA20 Disorders of newborn related to slow fetal growth or fetal malnutrition
LBW	P07 Disorders related to short gestation and LBW P07.0 ELBW P07.1 Other LBS P07.2 Extreme immaturity P07.3 Other preterm infants	KA21 Disorders of newborn related to short gestation or LBW, not elsewhere classified KA21.0 ELBW of newborn KA21.1 VLBW of newborn; KA21.2 LBW of newborn KA21.3 Extreme prematurity of newborn KA21.4 Preterm newborn

Abbreviations: ELBW, extremely low birth weight; HIV, human immunodeficiency virus; *ICD-10* and *ICD-11, International Statistical Classification of Diseases and Related Health Problems,*10th and 11th revisions; LBW, low birth weight; SGA, small for gestational age; VLBW, very low birth weight.

a *ICD-11* has additional codes for designating severity: XS25 for severe and XS0T for moderate.

b *ICD-10* rules default to B22.2 for children living with HIV infection with wasting, while these conditions are recorded separately in *ICD-11*, except when HIV infection wasting syndrome is the appropriate diagnosis in which case 1C62.3 can be used.

**Figure 1.**
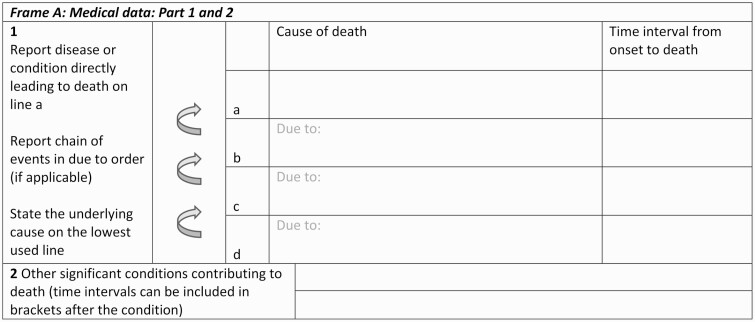
Codes from the *International Statistical Classification of Diseases and Related Health Problems, Tenth Revision* (*ICD-10*).

A constraint in using *ICD* codes is their lack of consistent alignment with current terminology used to describe undernutrition, and the rules are insufficiently nuanced to reflect current understanding of causes of undernutrition. For example, *ICD-10* rules for the E40–E43 undernutrition codes state that these codes are not be used in the following situations: intestinal malabsorption, nutritional anemia, starvation, or as a sequalae of protein-energy malnutrition [17]. Undernutrition is caused by multiple, often simultaneously occurring factors, including insufficient dietary intake, enteric dysfunction, and recurrent and persistent infections [18, 19]. Isolating the underlying determinants of undernutrition in an individual child is usually impossible. However, because *ICD-10* codes are the recommended standard for classifying conditions in the WHO International Form of Medical Certificate of Cause of Death (Figure 1), we tried to align as closely as possible to *ICD-10*.

The WHO death certificate medical portion has 2 sections. Conditions that directly led to death should be recorded in part 1 and listed in a causal chain. For example, if 3 conditions are listed in part 1, the immediate cause should be recorded on line 1a, the intermediate cause on line 1b, and the underlying cause on line 1c. Conditions that contributed to the death, but not directly in the causal chain, are listed in part 2 [17].

This guidance is intended for MITS and other postmortem studies, largely in LMICs, where clinical and health history are often limited. Owing to the physiological differences between neonates and older infants and children, the guidance is restricted to the postneonatal period (≥28 days).

### Objective 1: Establish Guidance for When to Include Severe and Moderate Wasting and Stunting in Death Certification and in Part 1 (Causal Chain) or Part 2 (Contributing Condition)

#### Severe Wasting in a Child Aged 1–59 Months With No History of LBW, Prematurity, or SGA

**Figure 2.**
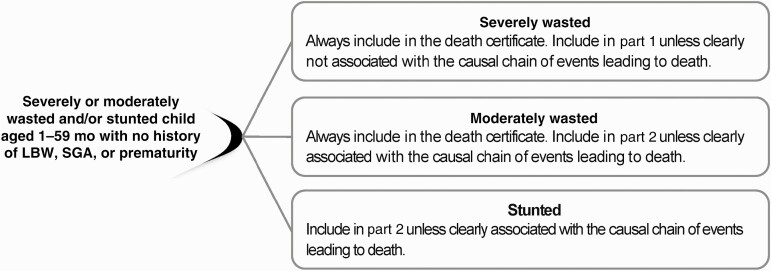
Wasting or stunting in a child aged 1–59 months with no history of low birth weight (LBW), small for gestational age (SGA), or prematurity.

First, if severe wasting criteria are met (Table 1), severe wasting should always be included in the death certificate in either part 1 or part 2; there are no circumstances where it should be excluded (Figure 2). Second, severe wasting should be included in part 1 unless it is clearly not associated with the causal chain of events leading to death, in which case it should be listed in part 2. *ICD-10* codes E40–E43 are most appropriate unless the child was HIV positive (in which case B22.2 should be used).

The rationale for these recommendations is as follows. Severe wasting is considered a medical emergency, is a well-established risk factor for child death, and is associated with extreme immunological, metabolic, and other physiological derangements [20, 21]. Under this extreme pathophysiological context, it would be unusual for severe wasting to *not* be part of the causal chain of events leading to death when present [1, 4]. In rare instances—such as sudden severe injury—severe wasting may not be an underlying cause of fatality. However, the extreme pathophysiology in severe wasting renders little capacity to tolerate and recover from any other insult, including infection, dehydration, and/or injury [20, 22–24]. Hence, in such circumstances, severe wasting should still be recorded as a contributing condition in part 2 of the death certificate.

The panel also considered scenarios whereby severe wasting status could be due to volume depletion as opposed to nutritional insufficiency (eg, if a child met severe wasting criteria based on low WLZ score but had a normal MUAC). Low WLZ score alone is a mortality risk factor even without low MUAC (and vice versa) [25]. However, in addition to nutritional insufficiency, dehydration (secondary to diarrhea or vomiting) and other causes of volume depletion (blood loss), can cause reductions in weight-for-length, albeit transiently.

The panel discussed the possibility of recommending recalculating WLZ scores in conditions that can cause volume depletion, such as to account for a potential 10% weight loss. The panel decided this was too hypothetical to make concrete recommendations at this time and noted that such a “correction factor” would only result in a category shift from severe to moderate wasting “at best.” However, the panel agreed that research is needed to determine the proportion of recalculated WLZ scores that would shift from below −3 to −3 or greater (in children without low MUAC) when potential volume loss is considered.

#### Moderate Wasting in a Child Aged 1–59 Months With No Known History of LBW, SGA, or Prematurity

First, if moderate wasting criteria are met (Table 1), moderate wasting should be always included in the death certificate in either part 1 or part 2; there are no circumstances where it should be excluded. Second, moderate wasting should be included in part 2 unless it is clearly associated with the chain of events leading to death (eg, serial measurements demonstrating rapid weight loss or decline in WLZ scores), in which case it should be recorded in part 1.

*ICD-10* code E44.0 is most appropriate unless the child was HIV positive (in which case B22.2 should be used).

#### Stunting in a Child Aged 1–59 Months

Stunting should be included in part 2 of the death certificate unless it clearly contributed to or was in the causal chain leading to death, in which case it would go in part 1. Circumstances under which this would occur are highly unlikely. The rationale for this recommendation is as follows. Approximately 2% of individuals in a population are expected to be stunted based on a normal distribution [15]. Risks related to stunting have been demonstrated at the population level (ie, when the population prevalence of stunting is high), it has been identified as a risk factor of poor outcomes (eg, increased mortality risk, delayed neurocognition) in the population, although the mechanisms behind these associations remain unknown. For these reasons, it is inappropriate to extrapolate stunting to the causal chain on an individual level [15].

We recommend that stunting—and other contributors to mortality risk, such as structural birth defects (eg, cleft palate), microcephaly, anemia, and sickle cell genotype—be systematically recorded in part 2 as contributing conditions, unless they are clearly part of the fatality causal chain (in which case they should be recorded in part 1). We also recommend further research to elucidate the role of poor linear growth in child fatality.

### Objective 2: Generate Guidance to Account for LBW, Prematurity, or SGA as Contributing or Causal Condition for Wasting in Deaths Among Infants Aged 1–3-Months

SGA, defined as birth weight (BW) for gestational age (GA) (BW-for-GA) below the 10th centile, is a prevalent and consequential form of undernutrition [1, 11, 26, 27]. InterGrowth or WHO fetal growth standards can be used to calculate BW-for-GA centiles [28, 29]. Classification of SGA requires accurate assessments of GA during pregnancy and weight at birth, both of which are often unavailable in low-resource settings, particularly precise estimates of GA, although this should become more widely available as prenatal ultrasonography scales up [30]. LBW, defined as <2500g at birth, can be caused by prematurity, SGA, or both. When accurate GA is unknown, LBW may be the only anthropometric proxy available. Young infants with LBW (whether due to prematurity and/or SGA) should exhibit sufficient catch-up growth by 4 months [31]. Hence, we focus on how documentation of LBW, prematurity, and SGA should be captured in relation to wasting status—the focus of this guidance—for deaths among 1–3-month-old infants.

#### Severe Wasting at Death and Known BW and GA

**Figure 3.**
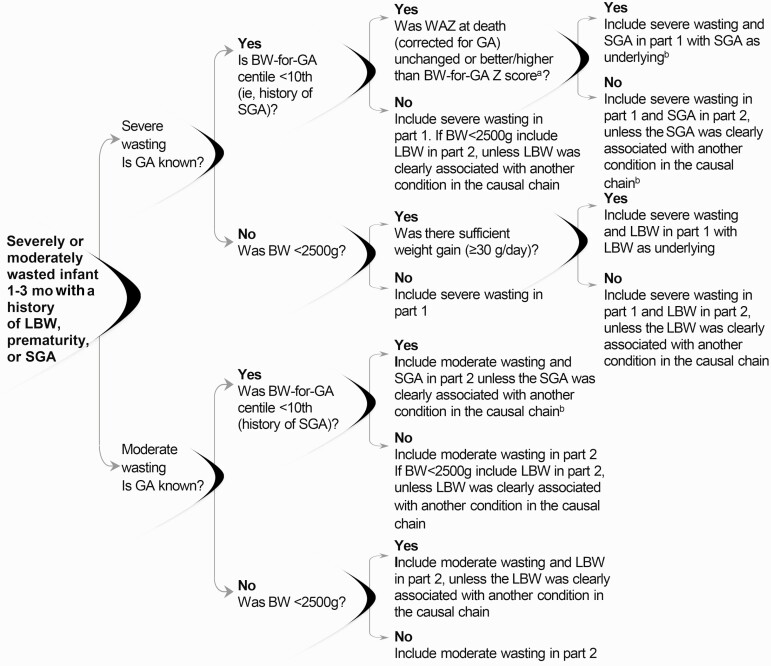
Moderate or severe wasting in children with a history of low birth weight (LBW), prematurity, or small for gestational age (SGA). As indicated in objective 1, severe wasting should be included in part 1 unless it is clearly not associated with the cause of death, in which case it should be listed in part 2. As indicated in objective 1, moderate wasting should be included in part 2 unless it is clearly associated with the cause of death, in which case it should be included in part 1. For severe or moderate wasting with birth weight (BW) for gestational age (GA) (BW-for-GA) below the 10th centile and concurrent prematurity, guidelines from the *International Statistical Classification of Diseases and Related Health Problems, Tenth Revision* (*ICD-10*) for coding prematurity should be followed. ^a^Alternatively was weight-for-age centile at death (corrected for GA) unchanged or better/higher than BW-for-GA centile? ^b^To allow for comparisons when GA is unknown, when BW is <2500g, LBW should also be included in part 2 (unless it was clearly associated with another condition in the causal chain, in which case it should be included in part 1).

Consistent with the guidance in objective 1, severe wasting should always be included in the death certificate and should be listed in part 1 unless it is clearly not associated with the causal chain of events leading to death (Figure 3).

#### BW-for-GA Below the 10th Centile (History of SGA)

First, calculate the WAZ score at death using GA-corrected age at death. Determine the BW-for-GA score to be able to compare the same metric (ie, *z* scores). Alternatively, calculate the weight-for-age centile at death to compare to the BW-for-GA centile. If the WAZ score (or weight-for-age centile) at death is unchanged or greater than (ie, better than) the BW-for-GA *z* score (or the BW-for-GA centile), severe wasting and SGA should be listed in part 1, with SGA as underlying severe wasting. Second, if the WAZ score at death corrected for GA is less than (ie, worse than) the BW-for-GA *z*, the severe wasting should be listed in part 1 and SGA in part 2 unless the SGA was clearly associated with another condition in the causal chain. In both circumstances, if the child was also preterm, follow *ICD-10* guidelines for recording prematurity in the death certificate. If BW was <2500g, LBW should also be included in part 2, unless clearly associated with another condition in the causal chain. This “dual coding” is to allow comparisons to scenarios where GA is unknown.

The rationale for these recommendations is as follows. Starting life SGA is an important cause of poor anthropometric status later in infancy [11]. If SGA is the primary cause of postneonatal wasting or underweight status, the WAZ score at death (corrected for GA) should not be lower than (worse than) the BW-for-GA *z* score [32–34]. However, if the anthropometric status of a young infant born SGA has deteriorated, the SGA is unlikely to be the predominant cause of the wasting (ie, another condition is contributing to the declining anthropometric status). Regardless of whether the WAZ score has improved since birth, if the child had severe wasting, this should still be included in the death certificate for the same reasons as stated in objective 1.

#### Severe Wasting at Death With a History of LBW but Unknown GA

Adequate weight gain, defined as ≥30g/d, should be used to assess whether the anthropometric status at death is worse than or better than at birth. If there was sufficient weight gain (an average of ≥30g/d), severe wasting and LBW should be included in part 1 with LBW as underlying the severe wasting. If there was insufficient weight gain (<30g/d average), severe wasting should be included in part 1 and LBW in part 2, unless clearly associated with another condition in the causal chain. The rationale for these recommendations is as follows. If an infant aged 1–3 months with a history of LBW showed adequate weight gain since birth, it is likely that LBW is the underlying cause of the severe wasting. If there is insufficient weight gain, it is likely that another condition is causing the severe wasting.

#### Moderate Wasting at Death With a History of LBW, Prematurity, or SGA

Consistent with the guidance in objective 1, moderate wasting should be listed in part 2 unless it is clearly associated with the chain of events leading to death. If GA is known and BW-for-GA was below the 10th centile, SGA should also be included in part 2 unless it was clearly associated with another condition in the causal chain. If the child was also preterm, follow *ICD-10* guidelines for recording prematurity in the death certificate. If BW was <2500g, LBW should also be included in part 2, unless clearly associated with another condition in the causal chain. This “dual coding” is to allow comparisons to scenarios where GA was unknown. If GA is unknown and the BW <2500g, LBW should also be included in part 2, unless it was clearly associated with another condition in the casual chain (see Figure 3).

### Objective 3: Standardize Methods for Using Postmortem Anthropometric Measurements to Identify Wasting and Stunting (Especially With Limited Antemortem Data)

#### Interpreting Postmortem Anthropometric Measurements

As in any study where anthropometrics represent important data points, standardized protocols and quality equipment must be used in postmortem studies as well to ensure accuracy and reliability [10, 35]. Recommended equipment includes (1) length boards with a fixed headpiece and a moveable foot piece perpendicular to the surface of the table on which the length board is placed; (2) digital scales with an approximate precision of 0.01kg and a taring function, calibrated regularly; and (3) nonstretchable, nontearable MUAC tape with 1-mm graduated precision. Personnel should be trained in standard procedures for conducting postmortem anthropometry (see Supplementary Material or CDC Micronutrient Survey Manual and Toolkit [36]). Repeated measurements and quality control measures, such as assessments for rounding error and digit preference, should be implemented.

Accurate and reliable anthropometric measurements depend on the use of standardized measurement procedures, appropriate and calibrated equipment, and data quality checks. Postmortem physiological changes, such as fluid shifts and rigor mortis, have the potential to affect postmortem, compared with antemortem, anthropometry. However, current evidence from MITS studies has shown that sufficient training, adherence to standardized procedures, use of appropriate regularly calibrated equipment, and data monitoring with feedback to measurers are the most important factors in ensuring accurate and reliable anthropometry during MITS [35]. Antemortem measurements, when available, and photographs taken as part of the standard MITS procedure, while vulnerable to subjectivity, can also be useful in confirmation of postmortem anthropometry and provide information on growth trajectories (eg, change in WLZ scores) over time. The panel recommends further research to correlate antemortem with postmortem anthropometric measurements to determine whether any postmortem correction factors are needed and also whether measurement of specific extremities can be used as a proxy for body length, because such assessments may be much easier than full body length to consistently and accurately measure [37–39].

#### Assessing Postmortem Edema

Based on the experience of pathologists conducting MITS, antemortem bipedal edema is persistent and can be evaluated postmortem, although a timeframe for doing so has not yet been established. Edema should be assessed during MITS by applying pressure to dorsal surface of both feet. If bilateral pitting edema is noted and not attributable to another condition (eg, congestive heart failure or nephrotic syndrome), the criteria for severe wasting are met, and per objective 1, severe wasting should be included in part 1 unless it is clearly not associated with the causal chain of events leading to death, in which case it should be listed in part 2.

Because there are limited published data correlating edema due to severe wasting in the antemortem and postmortem periods, the panel reviewed data from the Child Health and Mortality Prevention Surveillance (CHAMPS) network in drafting this guidance. For all CHAMPS cases with postmortem edema for which antemortem edema assessment was available (n = 29), the antemortem and postmortem assessments were concordant, with postmortem assessments all conducted within 24 hours of death (unpublished data). Therefore, there is a high likelihood that edema detected during MITS reflects antemortem physiological processes, rather than postmortem fluid shifts.

## CONCLUSIONS AND SUGGESTIONS FOR FUTURE RESEARCH

This guidance is intended for MITS and other postmortem studies, largely in LMICs, where clinical and health history are often limited. There were several potential areas for future research that emerged as part of the guidance development. First, acute dehydration or volume loss (including blood) before death can lead to reduced weight-based anthropometric measures (eg, WLZ score); to what degree is unknown. This can result in an overestimation of severe and moderate wasting due to nutritional status. The panel entertained potential methods to account for volume loss as part of postmortem anthropometrics, but in the absence of an evidence base for any specific method, the panel could not make recommendations.

Second, correlation between antemortem and postmortem anthropometric data, particularly length, are needed to understand the accuracy of postmortem measures and whether any correction factors need to be considered. Determinations of maximum postmortem interval, after which measurements or assessments of edema are unreliable, are also needed. Other measurements, such as long bone (eg, femur) length or digital imaging, may be easier and more reliable to accurately replicate; research is needed to determine whether such assessments could provide an accurate proxy for full body length.

Next, the panel focused on postneonatal deaths, in children <5 years old. There is a need for undernutrition classification in the neonatal period that can practically be applied in the context of postmortem studies in low-resource settings. And, although the evidence base on the magnitude of risk is limited, the risks associated with undernutrition, particularly severe wasting, persist beyond age 5 years. Additional research is needed to quantify the magnitude of risk of undernutrition in older children and adolescents, which can inform the development of similar guidance for integrating undernutrition into the casual chain of events leading to death in these populations.

In another potential research area, MITS studies provide opportunities to assess histopathological findings associated with micronutrient deficiencies and assess the role of this form of undernutrition in child mortality. MITS studies also offer unique and important opportunities to advance the understanding of the histopathophysiology of undernutrition. Three decades ago, autopsy-based studies of children with severe wasting provided evidence on the pathophysiology of undernutrition that still underpins current management practices [40, 41]. There has been a dearth of such data in the ensuing years. MITS offers an opportunity to renew this evidence base to identify improved and novel interventions for undernourished children and ultimately to reduce morbidity and mortality rates among this vulnerable population.

## Supplementary Data

Supplementary materials are available at *Clinical Infectious Diseases* online. Consisting of data provided by the authors to benefit the reader, the posted materials are not copyedited and are the sole responsibility of the authors, so questions or comments should be addressed to the corresponding author.

ciab851_suppl_Supplementary_Material_1

ciab851_suppl_Supplementary_Material_2

## Notes

***Acknowledgments.*** We thank Priya Gupta and Afrin Jahan for their review and analysis of Child Health and Mortality Prevention Surveillance (CHAMPS) network cases related to this guidance.

***Supplement sponsorship.*** This supplement is sponsored by MITS Surveillance Alliance Secretariat, led by RTI International, with funding from the Bill and Melinda Gates Foundation.

***Financial support.*** This work was supported by the MITS Surveillance Alliance and in whole or in part, by the Bill & Melinda Gates Foundation [OPP1180554]. Under the grant conditions of the Foundation, a Creative Commons Attribution 4.0 Generic License has already been assigned to the author accepted manuscript version that might arise from this submission.

***Potential conflicts of interest.*** All authors: No reported conflicts. All authors have submitted the ICMJE Form for Disclosure of Potential Conflicts of Interest. Conflicts that the editors consider relevant to the content of the manuscript have been disclosed.
