# Corrigendum to: Guidance for Systematic Integration of Undernutrition in Attributing Cause of Death in Children

**DOI:** 10.1093/cid/ciac084

**Published:** 2022-04-09

**Authors:** Christina R Paganelli, Nicholas Kassebaum, Kathleen Strong, Parminder S Suchdev, Wieger Voskuijl, Quique Bassat, Dianna M Blau, Donna M Denno

**Affiliations:** 1 RTI International, Seattle, Washington, USA; 2 Department of Health Metrics Sciences, University of Washington, Seattle, Washington, USA; 3 Department of Global Health, University of Washington, Seattle, Washington, USA; 4 Department of Anesthesiology and Pain Medicine, University of Washington Seattle, Washington, USA; 5 Department of Maternal, Newborn, Child and Adolescent Health and Aging, World Health Organization, Geneva, Switzerland; 6 Department of Pediatrics and Emory Global Health Institute, Emory University, Atlanta, Georgia, USA; 7 University of Amsterdam, Amsterdam, the Netherlands; 8 Amsterdam Centre for Global Health, Emma Children’s Hospital, Amsterdam University Medical Centres, Amsterdam, the Netherlands; 9 Amsterdam Institute for Global Health and Development, Amsterdam University Medical Centres, Amsterdam, the Netherlands; 10 The Childhood Acute Illness & Nutrition Network, Nairobi, Kenya; 11 ISGlobal, Hospital Clínic–Universitat de Barcelona, Barcelona, Spain; 12 Centro de Investigação em Saúde de Manhiça (CISM), Maputo, Mozambique; 13 ICREA, Pg. Lluís Companys 23, 08010 Barcelona, Spain; 14 Pediatrics Department, Hospital Sant Joan de Déu, Universitat de Barcelona, Esplugues, Barcelona, Spain; 15 Consorcio de Investigación Biomédica en Red de Epidemiología y Salud Pública (CIBERESP), Madrid, Spain; 16 Centers for Disease Control and Prevention, Atlanta, Georgia, USAand; 17 Department of Pediatrics, University of Washington, Seattle, Washington, USA

In the original publication of this article [Paganelli CR, Kassebaum N, Strong K, et al. Guidance for Systematic Integration of Undernutrition in Attributing Cause of Death in Children. Clin Infect Dis. Volume 73, Issue Supplement_5, 15 December 2021, Pages S374–S381, https://doi.org/10.1093/cid/ciab851], there were a number of minor errors which have been corrected in the originally published version of this manuscript. In Table 1 on the Moderate Wasting line, 'WLZ score below −2 but above −3' should read 'WLZ score below −2 but above or equal to −3'. In the MITS ANTHROPOMETRY section, the statement on WHO growth standards for children ≥5 years of age has been corrected to '>5 years of age'. In the section Objective 1: Establish Guidance for When to Include Severe and Moderate Wasting and Stunting in Death Certification and in Part 1 (Causal Chain) or Part 2 (Contributing Condition), the reference to Figure 1 has been corrected to reference Figure 2. The Figure 2 reference at the end of this section has been removed. The access date has been added to Reference 37.

