# Supplementary material for: Guidance for Systematic Integration of Undernutrition in Attributing Cause of Death in Children
Source: Clin Infect Dis. 2021 Dec 15;73(Suppl 5):S374–81. doi: 10.1093/cid/ciab851 (PMC8672773; doi:10.1093/cid/ciab851)
Supplement: ciab851_suppl_Supplementary_Material_1 [file ciab851_suppl_supplementary_material_1.docx]

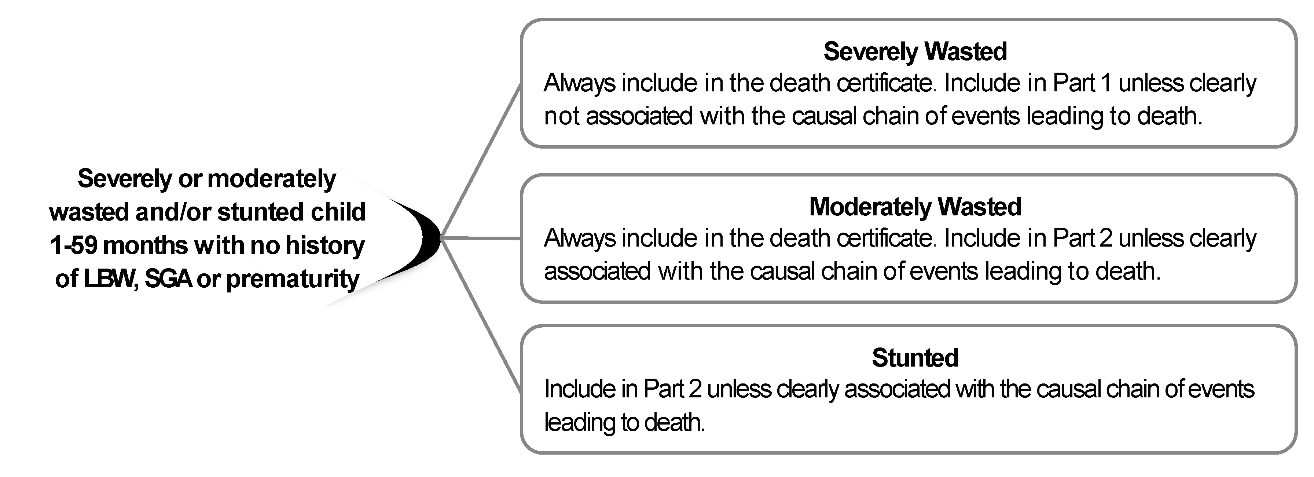





For severe or moderate wasting with birth weight (BW) for gestational age (GA) (BW-for-GA) below the 10th centile and concurrent prematurity, guidelines from the International Statistical Classification of Diseases and Related Health Problems, Tenth Revision (ICD-10) for coding prematurity should be followed.

a Alternatively was weight-for age centile at death (corrected for GA) unchanged or better/higher than BW-for-GA centile?

b To allow for comparisons when GA is unknown, when BW is <2500g also include LBW in Part 2 (unless it was clearly associated with another condition in the causal chain, in which case it should be included in Part 1).
