# Supplementary figures and images for: Guidance for Systematic Integration of Undernutrition in Attributing Cause of Death in Children
Source: Clin Infect Dis. 2021 Dec 15;73(Suppl 5):S374–81. doi: 10.1093/cid/ciab851 (PMC8672773; doi:10.1093/cid/ciab851)

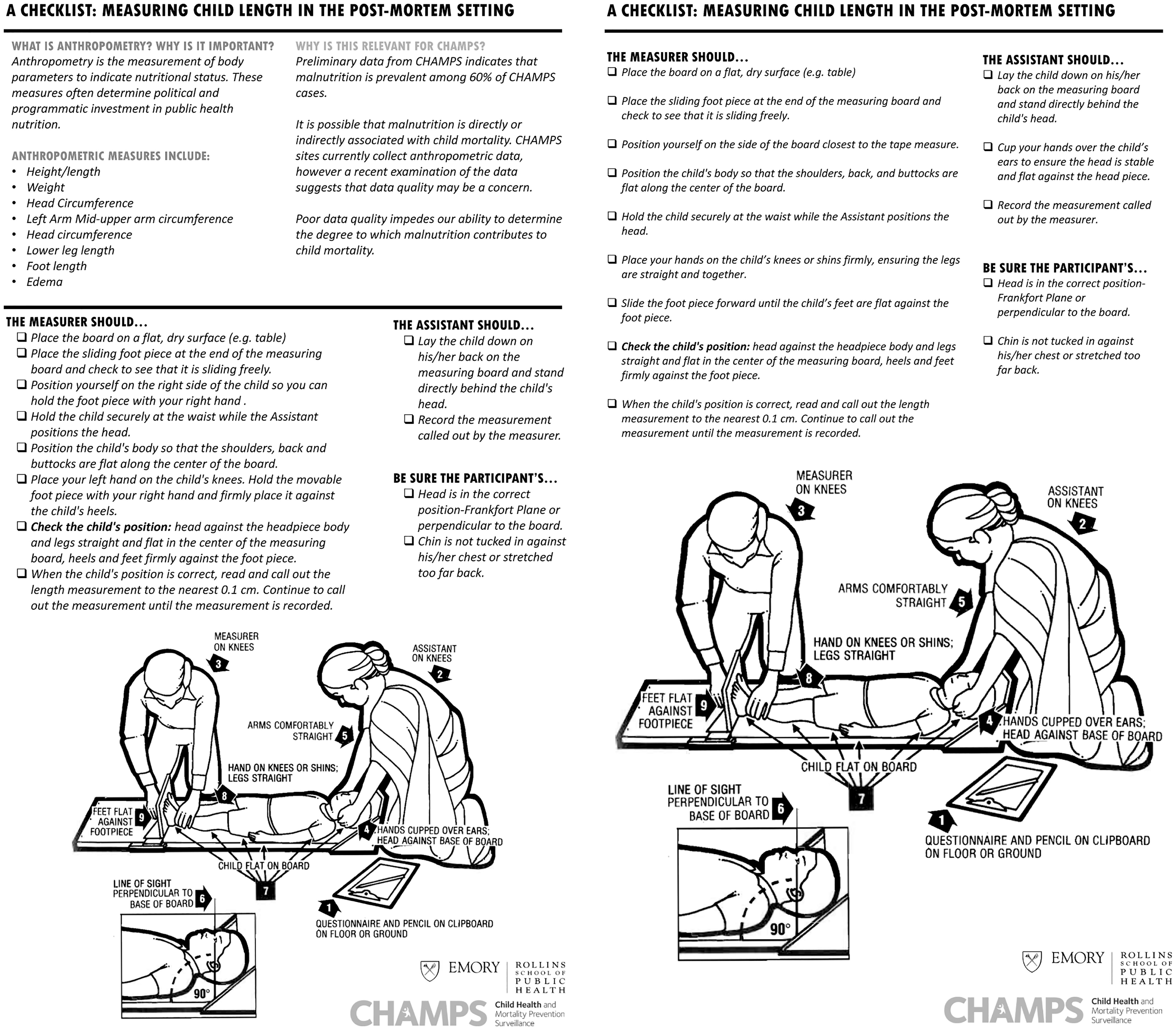

Supplement: ciab851_suppl_Supplementary_Material_2 [file ciab851_suppl_supplementary_material_2.jpeg]
